# Reinforced reasoning in medicine

**DOI:** 10.1111/jep.13269

**Published:** 2019-08-21

**Authors:** Daniel Auker‐Howlett, Michael Wilde

**Affiliations:** ^1^ Department of Philosophy, School of European Culture and Languages University of Kent Canterbury UK

**Keywords:** evidence‐based medicine, evidential pluralism, mechanisms, mechanistic reasoning, reinforced reasoning, virology

## Abstract

Some philosophers have argued that evidence of underlying mechanisms does not provide evidence for the effectiveness of a medical intervention. One such argument appeals to the unreliability of mechanistic reasoning. However, mechanistic reasoning is not the only way that evidence of mechanisms might provide evidence of effectiveness. A more reliable type of reasoning may be distinguished by appealing to recent work on evidential pluralism in the epistemology of medicine. A case study from virology provides an example of this so‐called *reinforced reasoning* in medicine. It is argued that in this case study, the available evidence of underlying mechanisms did in fact play a role in providing evidence in favour of a medical intervention. This paper therefore adds a novel and recent case study to the literature in support of evidential pluralism in medicine.

## 1 INTRODUCTION

A mechanism is a structure that performs some regular function by means of its component entities, activities, and their organization.^1^ A distinction is sometimes made between *evidence of underlying mechanisms* and *mechanistic evidence* for the effectiveness of a medical intervention (p123).^2^ Given a particular medical intervention, it is possible to have strong evidence of the underlying entities and activities involved in its mechanism of action without any evidence on this basis that the intervention is effective. However, *evidential pluralists* have argued that evidence of underlying mechanisms can sometimes provide mechanistic evidence, at least when the evidence of mechanisms is taken together with other types of evidence, for example, evidence of correlation from comparative clinical studies.^3^ Against this, some have argued that evidence of underlying mechanisms does not provide mechanistic evidence for the effectiveness of medical interventions. One such argument appeals to the unreliability of mechanistic reasoning, where mechanistic reasoning is the process of making a claim that an intervention will cause a particular effect on the basis of evidence of the underlying mechanisms. In this paper, we provide a response to a version of this argument that is provided by Miriam Solomon.^2^

Mechanistic reasoning is not the only way that evidence of underlying mechanisms might provide evidence for the effectiveness of a medical intervention. A more reliable type of reasoning may be distinguished by appealing to the recent work on evidential pluralism. In this paper, it will be argued that a case study from virology provides an example of this so‐called *reinforced reasoning* in medicine. It will also be argued that in this case study, the available evidence of underlying mechanisms did in fact play a role in providing evidence in favour of a medical intervention. This paper therefore adds a novel and recent case study to the literature in support of evidential pluralism in medicine.^3^, ^4^, ^5^, ^6^, ^7^, ^8^

## 2 MECHANISTIC REASONING AND MECHANISTIC EVIDENCE

A prominent criticism of evidence‐based medicine is that it downplays the research into pathophysiological mechanisms provided by the basic sciences.^9^ At best, this research figures on the lowest levels of the hierarchies ranking the quality of evidence for medical interventions (pp83‐87).^9^ Miriam Solomon has argued that there is a good reason to be skeptical about the role of basic science research in providing evidence for the effectiveness of medical interventions, namely, that “[t]here are countless examples of proposed interventions that make scientific sense and sometimes even work in vitro or in animal studies, but which turn out to be ineffective in humans” (p117).^2^ As an example, she talks about the false prediction that oestrogens would decrease the incidence of cardiac mortality, which was a prediction based upon the theory that oestrogens would lower the lipid concentration of the blood. Importantly, this was a false prediction based upon *mechanistic reasoning*, where mechanistic reasoning is the process of making a claim that an intervention will cause a particular effect on the basis of evidence of the underlying mechanisms (pp124‐130).^10^ (It is important to note that this definition of mechanistic reasoning requires *evidence* of the underlying mechanisms rather than simply a psychologically compelling *story* of a mechanism [pp349‐350].)^3^ Solomon thinks that this poor track record confirms that mechanistic reasoning does not provide evidence for the effectiveness of a medical intervention (p126).^2^ However, Solomon also recognizes the concern that downplaying the role of the basic sciences in evidence‐based medicine may result in overlooking an important source of information (pp119‐120).^2^ The question then is how to reconcile the supposed importance of the role of the basic sciences in evidence‐based medicine with the apparent low reliability of mechanistic reasoning.

One way to reconcile these lines of thought is to maintain that mechanistic reasoning fulfils an important *non‐evidential role* in a more complete epistemology of medicine than that articulated by current evidence‐based medicine. Let us call this *the non‐evidential approach to mechanistic reasoning*. This is the approach that is preferred by Solomon (p124).^2^ She thinks that mechanistic reasoning informed by basic science research is clearly important in medicine but that such reasoning provides no evidence for the effectiveness of a medical intervention. Given this, it must be that mechanistic reasoning is playing some other important but non‐evidential role. Solomon maintains that the important role for mechanistic reasoning is simply to *propose* medical interventions, which are then evaluated in terms of their effectiveness by the methods of evidence‐based medicine (p132).^2^ She says that “evidence‐based medicine should not discount mechanistic reasoning, unless it wants to bite the hand that feeds it” (p125).^2^

Solomon refers to the distinction between *the context of discovery* and *the context of justification*. This distinction is often traced back to the work of Hans Reichenbach.^11^ Broadly speaking, a scientific theory may be proposed by any method in the context of discovery, because it is evaluated in terms of its evidential standing in the separate context of justification. Solomon thinks that this distinction is helpful in providing an explication of the non‐evidential role for mechanistic reasoning in medicine. In particular, mechanistic reasoning is at home in the context of discovery, since it is a method by which a medical intervention may be proposed that need not thereby provide any justification for the effectiveness of that intervention. However, this is not a problem because there are also the methods of evidence‐based medicine, such as comparative clinical studies, that can evaluate the effectiveness of the proposed medical intervention in the separate context of justification. A more complete epistemology of medicine therefore finds a home for mechanistic reasoning within the context of discovery and a home for the methods of evidence‐based medicine within the context of justification, at least according to Solomon (pp124‐126).^2^ Given this, a distinct type of reasoning may be introduced by analogy to mechanistic reasoning: A comparative study may provide evidence of the existence of a correlation between an intervention and a positive health outcome, and *correlational reasoning* is the process of making a claim about the effectiveness of a medical intervention on the basis of such evidence of correlation. Indeed, Solomon says that “[h]ealth care interventions are judged effective when there is a correlation between the intervention and positive outcomes. Often it is not too much of a leap to infer that the intervention causes the positive outcome” (p117).^2^ This appeal to correlational reasoning gives the methods of evidence‐based medicine their proper role in providing evidence for the effectiveness of medical interventions without downplaying the methods of mechanistic reasoning from the basic sciences, which remain important in proposing potentially effective medical interventions.

Solomon acknowledges that the distinction between the context of discovery and the context of justification characterizes a romantic view of science. She says that “[s]ince the time of the logical empiricists, we have come to appreciate that the context of justification is not so rigorous, and that the context of discovery is not so unconstrained” (p125).^2^ Indeed, Daniel Steel also remarks that “[c]urrent discussions of the distinction in the philosophy of science literature take it as more or less given that aspects of the discovery process can be relevant to the assessment of hypotheses, and then proceed to consider the finer points of proposals about how this is so” (p97).^12^ In other words, the process by which a claim is proposed may be relevant to the level of justification of that claim. In particular, it may be that more informed mechanistic reasoning will lead to the proposal of a better justified claim about the effectiveness of a medical intervention. Indeed, Solomon herself says elsewhere that “the more we know about basic and other mechanisms, … the more likely we are to make accurate predictions and avoid drug failure by focusing on those interventions with the greatest probability of success” (p175).^2^

An alternative approach therefore maintains that certain instances of mechanistic reasoning can provide some evidence for the effectiveness of a medical intervention. Let us call this *the evidential approach to mechanistic reasoning*. This is the approach taken by Jeremy Howick.^10^, ^13^, ^14^ He acknowledges that some instances of mechanistic reasoning have led to harmful false predictions about the effectiveness of medical interventions (pp154‐157).^10^ However, Howick thinks that these false predictions were brought about by *low‐quality* mechanistic reasoning, because that reasoning was based upon insufficiently complete knowledge of the relevant mechanisms: “[M]echanistic reasoning based on partially understood mechanisms will not provide reliable evidence that an intervention caused a patient relevant outcome” (p139).^10^ Howick maintains that *high‐quality* mechanistic reasoning should be distinguished from low‐quality mechanistic reasoning, where mechanistic reasoning is high quality when it is based upon sufficiently complete knowledge of the relevant mechanisms and their behaviour under intervention (pp937‐938).^13^ He thinks that “if our knowledge of mechanisms is to count as reliable *evidence*, we need to know enough about the relevant mechanisms to predict how they will react to novel medical interventions” (pp129‐130).^10^ Of course, it is acknowledged that “there is much to stand in the way of mechanistic reasoning being of high quality since there are limits to our knowledge of bodily mechanisms and their interactions” (p939).^13^ However, Howick does also provide examples to suggest that “high‐quality mechanistic reasoning can provide reliable evidence that a treatment is effective” (p145).^10^

Solomon argues that this evidential approach conflates evidence of the underlying mechanisms with mechanistic evidence, where mechanistic evidence is any evidence about the effectiveness of a medical intervention that is provided by mechanistic reasoning (p123).^2^ She thinks that evidence of the underlying mechanisms does not provide mechanistic evidence that an intervention will be effective: “We could have strong evidence that the mechanisms operate, yet no evidence (or the weakest of evidence) that a particular proposed therapy will have the desired effect” (p123).^2^ Solomon gives the example of hormone replacement therapy: “We had strong evidence of hormonal effects on blood lipids, but weak (perhaps even no) evidence that this clinical intervention would reduce cardiac mortality, because we did not have full knowledge of the relevant complexity of the mechanisms” (p123).^2^ In other words, the reasoning based upon this evidence provided no mechanistic evidence that oestrogens would decrease the incidence of cardiac mortality. Her worry is that the complexity of pathophysiological mechanisms means that mechanistic reasoning does not determine the overall effect of a medical intervention because the reasoning is typically based upon incomplete evidence of the underlying mechanisms (pp131‐132)^2^:

A general problem with mechanistic accounts is that they are typically incomplete, although they often give an illusion of a complete, often linear, narrative. Incompleteness is the consequence of there being mechanisms underlying mechanisms, mechanisms inserted into mechanisms, background mechanisms that can fill out the mechanistic story, and mechanisms that can hijack regular mechanisms. That is, there is complex interaction of multiple mechanisms in a chaotic and multidimensional system. There are possible hidden mechanisms everywhere in mechanistic stories, despite an easy impression of narrative or causal completeness. Since we do not have a theory of everything, it is not possible to know in advance whether or not a particular mechanistic intervention will have the intended result.

Solomon even suggests that in the case of cystic fibrosis, as the evidence of the underlying mechanisms increased, this only served to stand in the way of providing mechanistic evidence for the effectiveness of an intervention because it revealed the high level of complexity of those mechanisms (pp126‐132).^2^ The idea is that such complexity means that evidence of underlying mechanisms will never be sufficiently complete to play a role in an instance of mechanistic reasoning that could provide any mechanistic evidence for the effectiveness of a medical intervention.

*Is this a good reason to think that evidence of underlying mechanisms cannot provide evidence for the effectiveness of a medical intervention?* At best, it has been shown that *standalone mechanistic reasoning* cannot provide evidence for the effectiveness of a medical intervention. However, evidence of underlying mechanisms may still provide evidence of effectiveness by playing a role in an alternative reasoning process. Indeed, *evidential pluralists* have argued that evidence of underlying mechanisms can provide mechanistic evidence for the effectiveness of a medical intervention, at least when it is taken together with other types of evidence, for example, evidence of correlation from comparative clinical studies.^3^, ^4^, ^5^, ^6^, ^7^, ^8^ Evidential pluralists emphasize the complimentary nature of evidence of mechanisms and evidence of correlation (p351)^3^:

Evidence of a linking mechanism helps show that the overall relationship between *A* and *B* is genuinely causal. But evidence of correlation helps to determine the net effect of a mechanism, and to show that it is not masked by further unknown mechanisms. Together, evidence of these two different things is very much stronger than evidence of one alone.

The basic idea is that each type of evidence comes with its own characteristic weakness for reasoning about the effectiveness of a medical intervention. On the one hand, thanks to the complexity of the underlying mechanisms, it can be difficult to determine the overall effect of a medical intervention on the basis of evidence of underlying mechanisms alone. On the other hand, it can be difficult to determine whether an observed correlation is causal on the basis of evidence of correlation alone. This is the familiar claim that correlation is not causation. However, evidence of an underlying mechanism that explains the extent of the observed correlation can help to address the characteristic weakness of evidence of correlation by making more plausible a causal interpretation of the correlation. And evidence of correlation helps to address the characteristic weakness of evidence of mechanisms by giving evidence of an overall effect.^5^ Clarke et al^3^ draw an analogy with reinforced concrete (p351):

[I]f steel is placed in concrete to produce reinforced concrete, we get a composite material where the concrete resists the compression and the steel resists the tension. The combination of two different materials produces a material that is much stronger than either of its components. In the same way, we argue that it is the combination of two different types of evidence which produces much stronger overall confirmation than would either type of evidence on its own. The important point is that this depends on the evidence being evidence of two types of things–correlations and mechanisms–that are different in character.

They say that “[t]ogether, evidence of these two different things is very much stronger than evidence of one alone” (p351).^3^ The point is that there are problems with reasoning about the effectiveness of a medical intervention on the basis of either underlying mechanisms or correlations alone. In other words, there are problems with both mechanistic and correlational reasoning. However, these problems may be addressed by adopting a type of evidential pluralism.

This evidential pluralism allows a novel type of reasoning in medicine to be introduced in contrast to both mechanistic and correlational reasoning. *Reinforced reasoning* is the process of making a claim about the effectiveness of a medical intervention on the basis of both evidence of a correlation and evidence of the underlying mechanisms. Importantly, it is plausible that reinforced reasoning does not share the weaknesses of mechanistic and correlational reasoning for drawing conclusions about the effectiveness of a medical intervention. In particular, this type of reasoning helps to address the characteristic weaknesses of both mechanistic and correlational reasoning by combining evidence of underlying mechanisms together with evidence of correlation. Reinforced reasoning therefore promises to be a process by which evidence of the underlying mechanisms may provide evidence in favour of the effectiveness of a medical intervention.

To sum up, it has been argued in this section that the present objection to mechanistic reasoning leaves untouched an alternative possible way that evidence of underlying mechanisms may provide evidence for the effectiveness of a medical intervention. Of course, it has not yet been shown that this alternative reinforced reasoning can in fact provide some evidence of effectiveness, that is, mechanistic evidence of effectiveness. In the next section, it will therefore be argued that a case study from virology provides a clear example of the differences between correlational, mechanistic, and reinforced reasoning in medicine. It will be argued that this case study demonstrates that evidence of mechanisms can in fact provide evidence for the effectiveness of a medical intervention by playing a role in an instance of reinforced reasoning.

## 3 A CASE STUDY IN VIROLOGY

Hepatitis C is a liver disease caused by infection from a single‐stranded, blood‐borne RNA flavivirus known as HCV.^15^ In some patients, an innate immune response is enough to clear this infection within a few months, whereas other patients go on to develop a *chronic* version of the infection. This chronic infection is a major cause of liver cancer and other diseases.^16^

A short time ago, the recommended optimal treatment for the chronic disease was a *combination therapy* of interferon alfa and ribavirin. Interferons are a family of cytokines that are a key component of the innate immune response against viruses. Interferons bind with receptors on the cell surface, leading to the expression of genes that prevent virus replication, facilitate viral clearance, and initiate the protection of neighbouring cells from further viral infection.^17^ However, viruses can produce accessory proteins to avoid or downregulate this immune response by inhibiting the expression of interferons.^18^ The idea is that this action may be countered by not relying solely on host expression and instead administering exogenous interferons.^19^ Indeed, a systematic review of randomized trials helped to establish that interferon monotherapy leads to an improvement in sustained virological response, defined as there being no detectable HCV RNA in blood tests after 6 months.^20^ Other trials helped to establish also that greater improvements in sustained virological response are achieved by combining interferons with a drug called ribavirin.^21^, ^22^ Ribavirin is a nucleoside analogue that is typically used as a broad‐spectrum antiviral based upon its demonstrated efficacy against viral replication for other viruses.^23^

Recently, there was a change in the recommended optimal treatment from the standard combination therapy to a *pegylated combination therapy* of peginterferon alfa and ribavirin, where pegylation aims to improve the therapeutic potential of the interferon by attaching to it a water‐soluble polymer called polyethylene glycol (PEG).^24^ It has been argued that this change in recommendation was the result of evidence for the increased effectiveness of pegylated combination therapy compared with the standard combination therapy.^16^ *What was the reasoning that justified this change in recommendation?*

A proponent of mechanistic reasoning may say that the change in recommendation was justified on the basis of evidence of the underlying mechanisms linking pegylated combination therapy to an improvement in rates of sustained virological response compared with the standard combination therapy. An acknowledged shortcoming of standard interferon therapy is that the antiviral activity of interferons is limited by the fact that they only remain in the body for a short amount of time.^25^, ^26^, ^27^ Indeed, even on three doses of interferon per week, there will be two days of the week in which there is no clinically relevant drug concentration (p230).^25^ This is a particular problem given the rapid rate of replication of HCV.^28^ However, there is good evidence of a couple of mechanisms by which pegylation prolongs the *biological half‐life* of interferons, that is, the time it takes biological processes to remove half of the substance.^27^, ^29^, ^30^ In particular, pegylation prolongs the half‐life of the interferon by increasing its effective size, thereby decreasing renal and cell clearance. In addition, pegylation disrupts enzyme activity, which also prolongs the half‐life of the interferon by decreasing proteolysis. In turn, this prolonging of half‐life increases the bioavailability of interferons, potentially improving antiviral activity by permitting more sustained pressure on the virus. Indeed, clinically significant serum levels of peginterferon have been detected up to a week after administration.^30^ On the basis of this evidence of the different pharmacokinetic effects of pegylated and standard combination therapy, the proponent of mechanistic reasoning may claim that there is mechanistic evidence for the greater effectiveness of pegylated combination therapy compared with standard combination therapy.

However, this standalone mechanistic reasoning plausibly provides no mechanistic evidence that pegylated combination therapy is more effective than the standard combination therapy at improving rates of sustained virological response. Although there is evidence of the underlying mechanisms linking pegylated combination therapy to increased antiviral activity, there is the same worry as discussed above, namely, that the complexity of the relevant mechanisms is not well understood. In particular, there may be other unknown, complicating mechanisms that make it difficult to determine any overall increased beneficial effect for pegylated combination therapy. For example, a standard problem for pegylated therapeutics is that increasing the size of a protein through pegylation has been shown to reduce its biological activity by disrupting the ability of the protein to bind with the relevant receptor.^29^ It may be that this reduction in biological activity will outweigh any additional beneficial effect due to pegylation. It is for this reason that it is widely acknowledged that “the net biologic activity of each particular PEGylated product may be difficult, if not impossible to predict” (p33).^27^

Of course, the fact that this standalone mechanistic reasoning did not provide mechanistic evidence for the increased effectiveness of the pegylated combination therapy is unlikely to come as a surprise to the critic of the evidential approach to mechanistic reasoning: It is simply another case where incomplete knowledge of the complexity of the underlying mechanisms precludes providing evidence of an overall beneficial effect. The case provides further evidence that mechanistic reasoning is at home in the context of discovery rather than the context of justification. Instead, the critic might recommend looking towards evidence of correlation in order to provide this evidence of a beneficial effect, where this evidence of correlation is typically obtained from comparative studies (pp124‐125).^2^ They might say that the change in recommendation from standard to pegylated combination therapy was based upon evidence of a correlation between pegylated combination therapy and improved rates of sustained virological response. In other words, the change in recommendation was justified on the basis of standalone correlational reasoning.

Unfortunately, it also looks like the change in recommendation was not justified solely on the basis of correlational reasoning. A number of randomized trials had concluded that pegylated combination therapy was *correlated* with a statistically significant improvement in rates of sustained virological response when compared with the standard combination therapy.^31^, ^32^, ^33^ A later systematic review of 27 randomized clinical trials agreed that pegylated combination therapy is *correlated* with a significant improvement in rates of sustained virological response.^34^ However, the question is whether this correlation is explained by the increased *causal effectiveness* of the pegylated combination therapy compared with the standard combination therapy. It may be that there are more plausible alternative explanations of the observed correlation, such as bias in the available trials. Indeed, the systematic review also concluded that there was only *moderate evidence* of the increased effectiveness of pegylated combination therapy (p23).^34^ All trials included in the review were considered to have a high risk of bias, that is, a propensity to overestimate the benefits of the intervention (p3).^34^ In addition, the only blinded randomized trial found no increased effectiveness for pegylated combination therapy.^35^ Given this, standalone correlational reasoning in this case at best provided only moderate evidence for the increased effectiveness of pegylated combination therapy, and this moderate evidence does not seem to be enough to properly justify the change in recommendation to pegylated combination therapy.

Although neither standalone correlational reasoning nor standalone mechanistic reasoning can justify the change in recommendation from standard to pegylated combination therapy, it should not be concluded that there was insufficient justification for this change in recommendation. It might be that the change in recommendation was justified on the basis of an instance of *reinforced reasoning*. Although the available evidence of correlation at best provides only moderate evidence for the increased effectiveness of pegylated combination therapy, it might be that this evidence is more conclusive when it is taken in combination with the available evidence of underlying mechanisms. In particular, the evidence of a correlation between pegylated combination therapy and improved rates of sustained virological response is inconclusive because it may be explained by bias in the available trials.^34^ However, evidence of the underlying mechanisms makes it more plausible that this correlation is explained by the increased causal effectiveness of pegylated combination therapy, because there is evidence of a mechanism by which pegylation improves the antiviral activity of interferons by prolonging their biological half‐life. In effect, this evidence of the underlying mechanisms can help to rule out bias as the explanation of the observed correlation (pp343‐346).^3^

It might be objected that it is illegitimate to rely upon evidence of mechanisms to help rule out bias as an alternative explanation of the observed correlation. It has already been shown that standalone mechanistic reasoning is unreliable in this case because it cannot determine an overall beneficial effect: It may be that any additional beneficial effect brought about by increasing the bioavailability of the interferons is outweighed by the reduction in biological activity brought about by disrupting the binding potential of the interferons. However, this problem for the reliability of standalone mechanistic reasoning is not a problem for the reliability of reinforced reasoning, because reinforced reasoning relies also upon evidence of correlation, and this evidence of correlation gives evidence of an overall beneficial effect. In particular, the available systematic review shows a robust correlation between the pegylated combination therapy and improved rates of sustained virological response.^34^ In other words, in an instance of reinforced reasoning, it is not just the evidence of mechanisms that reinforces the correlational reasoning, the evidence of correlation also reinforces the mechanistic reasoning (pp341‐351).^3^

To sum up, there is some good evidence of the underlying mechanisms linking pegylated combination therapy to improved rates of sustained virological response. But standalone mechanistic reasoning on this basis provides no evidence for the increased effectiveness of pegylated combination therapy, because the complexity of the underlying mechanisms makes it impossible to determine the overall effect of the pegylated therapy. Of course, evidence of correlation can help to better determine such an overall effect. Unfortunately, standalone correlational reasoning in this case also provides insufficient evidence for the increased effectiveness of pegylated combination therapy. Although there was moderate evidence that the pegylated combination therapy is correlated with improved rates of sustained virological response compared with the standard combination therapy, there was still a possibility that this correlation was best explained as a result of bias in the available trials. However, this explanation looks less plausible in the light of the evidence of the underlying mechanisms linking pegylation with improved antiviral activity. Reinforced reasoning therefore provides more conclusive evidence of the increased effectiveness of pegylated combination therapy. Importantly, this instance of reinforced reasoning provides greater evidence for the increased effectiveness of pegylated combination therapy than standalone correlational reasoning. Moreover, this difference between standalone correlational reasoning and reinforced reasoning is the addition of evidence of mechanisms. This shows that evidence of underlying mechanisms can provide some evidence in favour of the effectiveness of a medical intervention.

## 4 CONCLUSION

Reinforced reasoning involves making claims about the effectiveness of a medical intervention on the basis of both evidence of a correlation and evidence of the underlying mechanisms. In this paper, it has been argued that a case study from virology provides an instance of such reinforced reasoning. It has also been argued that reinforced reasoning is one way in which evidence of underlying mechanisms can provide evidence for the effectiveness of a medical intervention. Much of the literature on evidential pluralism concerns historical case studies.^8^ This paper adds a novel and recent case study to the literature in support of evidential pluralism in medicine.

## FUNDING INFORMATION

Daniel Auker‐Howlett: Arts and Humanities Research Council Consortium for Humanities and Arts South‐East England (CHASE) Doctoral Training Partnership (AH/L503861/1).

Michael Wilde: Arts and Humanities Research Council Project: Evaluating Evidence in Medicine (AH/M005917/1).

## CONFLICT OF INTEREST

The authors declare no conflict of interest.
